# Sacroiliac joint tuberculosis: surgical management by posterior open-window focal debridement and joint fusion

**DOI:** 10.1186/s12891-017-1866-9

**Published:** 2017-11-29

**Authors:** Guo Zhu, Li-Yuan Jiang, Zhang Yi, Li Ping, Chun-Yue Duan, Cao Yong, Jin-Yang Liu, Jian-Zhong Hu

**Affiliations:** 1 Department of Spine Surgery, Xiangya Hospital Central South University, Changsha, China; 2 Key Laboratory of Organ Injury, Aging and Regenerative Medicine of Hunan Province, Hunan, China

**Keywords:** Sacroiliac joint tuberculosis, Focal debridement, Joint fusion

## Abstract

**Background:**

Sacroiliac joint tuberculosis(SJT) is relatively uncommon, but it may cause severe sacroiliac joint destruction and functional disorder. Few studies in the literature have been presented on SJT, reports of surgical treatment for SJT are even fewer. In this study, we retrospectively reviewed surgical management of patients with severe SJT of 3 different types and proposed to reveal the clinical manifestations and features and aim to determine the efficiency and security of such surgical treatment.

**Methods:**

We reviewed 17 patients with severe SJT of 3 different types who underwent posterior open-window focal debridement and bone graft for joint fusion. Among them,five patients with anterior sacral abscess had anterior abscess curettage before debridement. Two patients with lumbar vertebral tuberculosis received one-stage posterior tuberculous debridement, interbody fusion and instrumentation. Follow-up was performed 36 months (26 to 45 months) using the following parameters: erythrocyte sedimentation rate(ESR), status of joint bony fusion on CT scan, visual analogue scale (VAS) and the Oswestry Disability Index (ODI).

**Results:**

Buttock pain and low back pain were progressively relieved with time. 6 months later, pain was not obvious, and ESR resumed to normal levels within 3 months. Solid fusion of the sacroiliac joint occurred within 12 months in all cases. No complications or recurrence occurred. At final follow-up, all patients had no pain or only minimal discomfort over the affected joint and almost complete functional recovery.

**Conclusions:**

Posterior open-window focal debridement and joint fusion is an efficient and secure surgical method to treat severe SJT. If there is an abscess in the front of the sacroiliac joint, anterior abscess curettage should be performed as a supplement.

**Electronic supplementary material:**

The online version of this article (10.1186/s12891-017-1866-9) contains supplementary material, which is available to authorized users.

## Background

Bones and joints infected with tuberculosis (TB) are relatively uncommon, accounting for approximately 2–5% of all TB cases in Europe and the USA and approximately 10–15% of extrapulmonary tuberculosis (EPTB) cases. However, the incidence may rise to 15–20% in undeveloped countries. Especially in Asia,the number may be even higher [1]. Among osteoarticular tuberculosis, approximately 50% of cases are involved in the spine, mainly the thoracic and lumbar segments. Sacroiliac joints infected with tuberculosis are rare, comprising approximately 5–10% of cases with skeletal tuberculosis [2–4].

Moreover, SJT occurs in a concealed manner and progresses slowly. It is frequently misdiagnosed due to its nonspecific manifestation and syndromes [4]. In addition, its rare appearance usually causes it to be overlooked and diverts attention toward other similar, common diseases. The duration between the onset of initial symptoms and definite diagnosis is usually 16–19 months. Delayed and inappropriate treatments occur frequently and cause remarkable pain, extensive destruction and instability of the sacroiliac joint. By that time, surgery is essential [5]. There are only a few studies that have reported about surgical treatment and the types of cases were simple and lacked variation [6–10]. Moreover, neither details of the surgical procedure nor post-surgical CT scans of follow-up were well presented. Therefore, we retrospectively reviewed 17 surgical cases that covered 3 types of sacroiliac tuberculosis in our classification and try to fill up the deficiency of previous studies.

## Methods

We reviewed 17 patients with severe SJT (8 males and 9 females, II,III or IV type of Kim’s classification [10]) who were managed surgically in our department from July 2005 to February 2014. The average age of the patients was 30.5 years, ranging from 18 to 57 years. According to Kim’s classification, there were 7 type II cases, 4 type III cases and 6 type IV cases. All patients were infected unilaterally in the sacroiliac joint, 8 left and 9 right (Table 1). The mean duration from initial symptoms to final diagnosis was 16.2 weeks, ranging from 6 weeks to 15 months.

**Table 1:** Patient data

Patients NO.	Gender	Involved side	Other infected side	Kim’s type	Classificatin of our study
1	F	L	–	III	A
2	F	R	–	II	A
3	M	R	Lung	IV	B
4	F	L	Lung	II	A
5	F	L	–	IV	B
6	F	L	–	III	A
7	F	R	–	II	A
8	M	L	–	IV	B
9	M	R	–	IV	B
10	M	R	–	II	A
11	M	L	Lumbar	IV	C
12	M	L	Lung	III	A
13	F	R	Lumbar	IV	C
14	F	R	–	III	A
15	M	L	–	II	A
16	M	R	–	II	A
17	F	R	Lung	II	A

Low back pain and buttock pain were the biggest complaints in all 17 patients. Five of the patients had difficulty walking and could not totally bear weight. Four patients suffered from sciatica, but the pain was confined to the buttocks or thigh, and the cruses were not affected. Other nonspecific symptoms were found, such as fever in 12 patients, sweating in 8, poor appetite in 6, and weight loss in 2. There were 4 patients with a palpable inguinal mass, and 1 of them developed a groin sinus tract after a pre-hospital needle biopsy. Four patients also had pulmonary tuberculosis. One patient had been diagnosed as having type 2 diabetes for 4 years. The physical examination indicated that all patients had tenderness over the sacroiliac joint; Patrick’s test and pelvic compression tests were positive. Passive motion of the lesioned joint in extreme flexion and extension was limited and painful.

The primary diagnosis of SJT was guided by the above symptoms and the following laboratory findings: routine blood examination (Blood RT), erythrocyte sedimentation rate (ESR), tuberculin purified protein derivative (PPD) test, T-SPOT.TB test, and anti-tuberculosis antibodies. Diagnosis was also assisted by imagology findings including an anteroposterior view pelvic X-ray, sacroiliac joint CT scans and MRI. The final diagnosis was based on pathological biopsy results or mycobacterium culture.

Once the preliminary diagnosis of tuberculous sacroiliitis was made, isoniazid 5 mg/kg/d, rifampicin 10 mg/kg/d, ethambutol 15 mg/kg/d, and pyrizinamide 25 mg/kg/d were orally administered in combination. Before surgery, anti-tuberculosis chemotherapy and nutritional support therapy was performed simultaneously for 7 to 14 days, until patients resumed normal temperature, ESR decreased significantly, and anemia and hypoproteinemia were rectified.

Based on surgical methods and radiological manifestations, we classified the cases (Kim’s classification type II, III or IV) into 3 types. Type A was defined as severe destruction of the sacroiliac joint, with or without a posterior abscess of sacroiliac joint. Type B was defined as severe destruction of the sacroiliac joint, with an anterior abscess of sacroiliac joint, with or without a posterior abscess. Type C was defined as severe destruction of the sacroiliac joint accompanied by spinal tuberculosis.

**Fig. 1:**
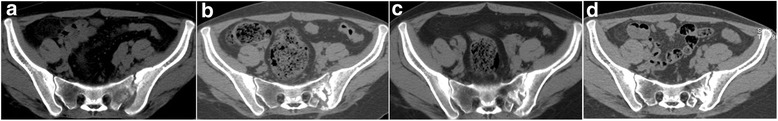
CT of patient NO.1 pre- and postoperatively at 3, 6 and 12 months. **a** Bone destruction and cyst on the sacrum side. **b** Bone fusion on the sacrum side as early as 3 months after surgery. **c** More evident bone fusion on both sides of the joint 6 months after surgery. **d** Solid joint fusion of the sacroiliac joint 12 months after surgery

**Fig. 2:**
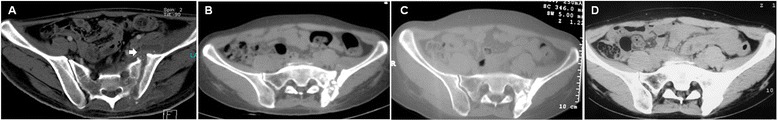
CT of patient NO.8 pre- and postoperatively at 3, 6 and 12 months. **a** Severe bone destruction on both sides of the joint; arrow indicates a rupture in the front of the sacroiliac joint capsule, with a sinus tract connecting to the abscess in the iliopsoas. **b** Ample bone graft in the lesion side and no obvious bone fusion 3 months after surgery. **c** Bone graft remodeling and no occurrence of bone absorption. **d** Solid joint fusion within 12 months after surgery

**Fig. 3:**
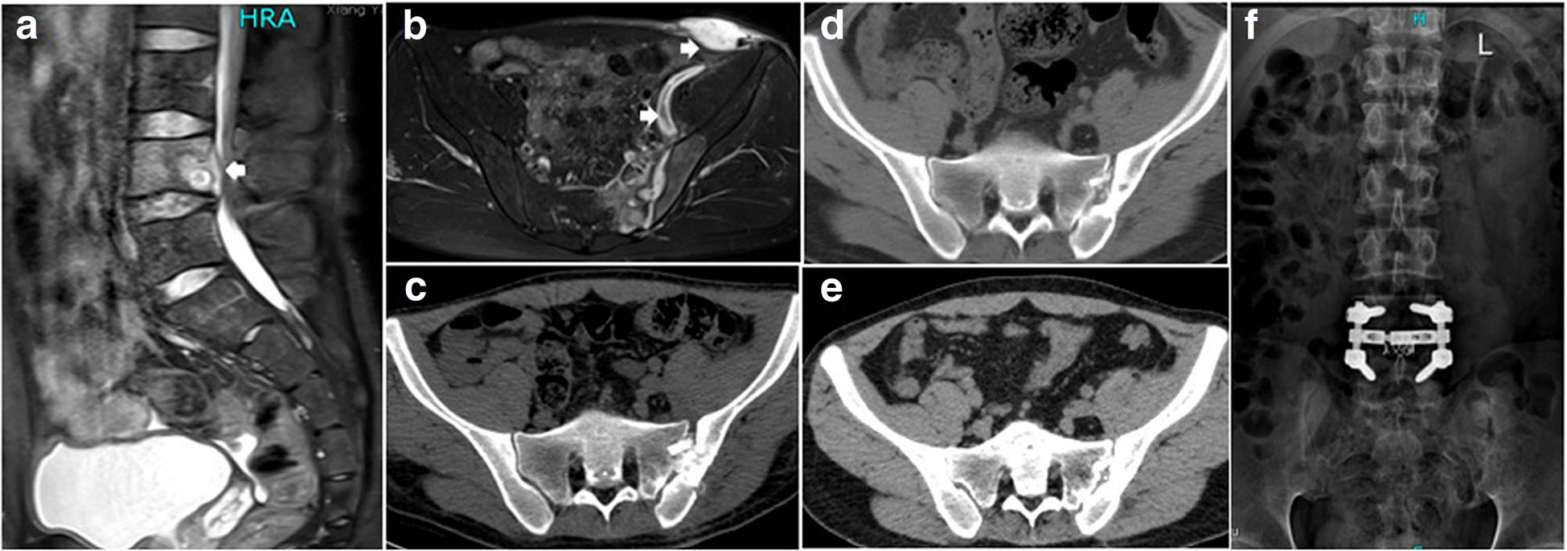
MRI and CT of patient NO.11 pre- and post-operation of 3,6 and 12 months and X-ray of 12 months after surgery. **a** L4 vertebra was eroded and epidural sac was compressed (**b**)Arrow indicates a inguinal abscess with a sinus track attaching to iliopsoas abscess. **c**-**e**)Procession of sacroiliac joint fusion at 3,6 and 12 months postoperation. Solid joint fusion was found within 12 months postoperation. (**f**)X-ray indicated solid joint fusion and good instrumentation

According to this classification, 11 type A patients (4 male and 7 female) underwent posterior open-window focal debridement, bone graft and joint fusion (Fig. 1). Four type B patients (3 male and 1 female) received anterior abscess curettage before posterior open-window focal debridement and joint fusion (Fig. 2). Two type C patients (1 male and 1 female) with lumbar tuberculosis underwent posterior open-window focal debridement, joint fusion and one-stage posterior lumbar focal lesion clearance,interbody fusion and internal fixation. One of the 2 type C patients also had an anterior abscess and received anterior abscess clearance first (Fig. 3 and Additional file 1: Figure S4).

The surgical management was performed using a posterior approach as follows: The patients were kept in prone position, and an 8–10 cm arc incision was made, centered over the posterior superior iliac spine to expose the sacroiliac joint. A 3 × 4 cm bone window was chiseled at the corresponding position of the lesion. The focus of tuberculous was completely curetted through to the healthy bleeding bone. The sacroiliac joint was irrigated with saline, 0.2 g isoniazid and 1.0 g streptomycin powder were dusted. Healthy bone was acquired from the iliac crest and transplanted into the cavity of the bone defect. If the bone block curetted out was not eroded, it could be replaced after appropriate modifications. Finally, a drain tube was placed and the incision was closed in layers. The drain tubes were removed 3 days after surgery.

For type B patients and one of type C patients, anterior abscess curettage was applied before posterior approach surgeries. A 3- to 4 cm incision was made from the anterior superior iliac spine to the symphysis pubis. The iliopsoas were exposed extraperitoneally and the sinus ostium was enlarged along the sinus tract to the abscess. If there was no sinus ostium, the relative position of abscess was determined, avoiding the ureter and iliac vessels. The abscess was then dissected bluntly. Pus and caseous necrotic tissue were scraped out, and the area was irrigated repeatedly until it was thoroughly clean. Then 100 mg isoniazid and 500 mg streptomycin powder were dusted and a drainage tube was placed, which was removed 3 days after surgery.

For type C patients, one-stage posterior lumbar tuberculous debridement, interbody fusion and instrumentation was applied as previously reported [11]. The general pictures in operation were showed in Additional file 1: Figure S4.

The same quadruple anti-tuberculosis chemotherapy extended 12 to 18 months postoperatively. Hepatic and renal function were monitored monthly in case of any adverse reactions to the antituberculosis drugs. No external fixation was needed except for 2 type C cases, which needed immobilization in the lumbar vertebral segment. Out-of-bed activities on crutch were allowed 3 months after surgery. Patients were allowed full weight ambulation 6 months postoperatively. The average follow-up was 36 months (26 to 45 months). The parameters used in follow-up were ESR, status of joint bony fusion on CT scan, VAS and ODI [12–14]. Data of VAS and ODI of pre-operation, 3-,6-, 12- and 24 month were analyzed using SPSS software (version 13.0, Chicago, IL, USA) with the Wilcoxon rank sum test and Mann–Whitney test. The threshold for statistical significance was set at *P* value ≤0.05.

## Results

Severe destruction of the sacroiliac joint was indicated on CT scans: broadened joint gaps or cystic changes of different extents, bone erosion of various ranges on the sacrum or iliac side, and diverse amounts of sequestra were found. In the operations, necrotic tissue and sequestra presented in all cases. Four type B patients had a rupture in the front of the sacroiliac joint capsule, which led to abscess formation in the iliopsoas. One of the type C patients (patient No.11) formed an inguinal abscess with a sinus tract attaching to the iliopsoas abscess. Additionally, the L4 vertebra was eroded and the epidural sac was compressed (Figs. 3a-b). The final diagnosis of all patients was confirmed by pathological biopsy results or mycobacterium culture.

The average time of surgery was 112.6 min (range, 92 to 158 min) and the average amount of estimate blood loss was 187.6 mL (range, 80 to 350 mL) (Table 2). The data of lumbar surgical management was not included in the calculation. No side injuries occurred in any patients. The mean preoperative ESR was 52.2 mm/h (24 to 92 mm/h), which decreased to 36.5 mm/h (23 to 53 mm/h) 7 days after surgery and returned to normal (9.3 mm/h, 6 to 14 mm/h) within 3 months in all patients. Bone fusion could be observed on CT scan as early as 3 months postoperatively (Fig. 1b) and was more evident at 6 months (Figs. 1c, 2c). All patients had solid joint fusion within 12 months (Fig. 1d, Fig. 2d, Fig. 3e-f). At last follow-up, all patients were completely weight bearing and had no pain when walking, going upstairs or bearing a heavy load. VAS and ODI scores decreased remarkably at 3 and 6 months after surgery. Twelve months after surgery, there was no pain or only minimal discomfort on the lesion side, and function was almost completely recovered. VAS and ODI scores of each patients (*n* = 17) had significant differences at 3-, 6-, 12 months postoperative compared to one time point before respectively (*P* < 0.001) and there had no statistically significant differences between 12 and 24 months postoperative (*P* = 0.77 and 0.23, respectively, Table 2). Between patients with abscess, sinus or lumbar tuberculosis (type B and C, *n* = 6) and the rest patients (*n* = 11), VAS scores had no statistically significant differences pre- and post-operation(pre-, *P* = 0.41; post- 3 months, *P* = 0.22; post- 6 months, *P* = 0.24; post- 12 months, *P* = 0.27; post- 24 months, *P* = 0.91). ODI scores had statistically significant differences only at 6 months postoperation (*P* = 0.01) and there was no statistically significant differences at other time point (pre-, *P* = 0.37; post- 3 months, *P* = 0.08; post- 12 months, *P* = 0.37; post- 24 months, *P* = 0.54). At the final follow-up, no recurrences had occurred.

**Table 2:** Outcome of 17 patients received surgical management

Outcome	Surgical management and joint fusion(*n* = 17)				
Estimate Blood Loss(ml)	187.6 ± 86.7				
Duration of Surgery(min)	112.6 ± 18.5				
	Pre-op		Post 7 day		Post 3 month
ESR(mm/h)	52.2 ± 23.0		36.5 ± 8.8		9.3 ± 2.7
	Time Postoperation				
	Pre-op	3	6	12	24
VAS	6.0 ± 1.7	3.0 ± 0.8	2.1 ± 0.6	0.5 ± 0.5	0.5 ± 0.5
ODI (%)	62.7 ± 21.4	49.7 ± 11.8	35.3 ± 11.3	3.1 ± 1.7	2.7 ± 1.6

The normal reference value of ESR in our hospital: <15 mm/h (male), <20 mm/h (female)

## Discussion

SJT is uncommonly encountered, especially in developed countries, there were only a few scattered reports. The relatively low incidence and lack of experiences of doctors with SJT which may make it easily misdiagnosed. In addition, the intrinsic natures of SJT may be another reasons for its misdiagnosis and cause the delayed presentation of patients. a). SJT occurred in a concealed manner and the symptoms are nonspecific in the early phase. That may be attributed to sacroiliac joints being amphiarthrodial joints with limited range of motion. Their main function is to stabilize the joints, transfer and dissipate truncal loads to the lower limb. Also in the early stage, the pathologic changes are mainly confined in the synovium and fibrocartilage [5]. On account of the relative stability of the joints, the functional loss is not evident. b). Because of the mixed innervation of the sacroiliac joint and lumbosacral spine,SJT may mimic various pathologies; for example, myofascitis, lumbar instability, lumbar intervertebral disc herniation, or ankylosing spondylitis et al. Inappropriate treatment and delayed diagnosis may involved. [15, 16]. c). The course of the SJT is slow and progressive and it failed to draw adequate attention of some of the patients or even doctors which may lead to delayed treatments. When patients present with significant pain, difficulty walking or visible abscess formation, the sacroiliac joint usually has severe destruction.

CT and MRI are more sensitive to early subtle changes and can indicate the range and extent of the lesions [17, 18]. Especially for MRI, it is the best imaging tool for presenting the affected soft tissues which make it ideal method to detect tuberculous abscess [19]. However, the diagnosis of SJT should be confirmed by pathologic biopsy or culture for mycobacteria after surgery.

According to the severity of radiological features, Kim’s classification separates SJT into 4 types. Patients with severe destruction of joint, mild cystic changes or instability of the sacroiliac joint are recommended for surgical procedures. Because of the drainage of pus,curettage for necrotic tissue and arthrodesis may help acquire early bone fusion [10].

According to surgical managements and radiological findings,we classified the cases into 3 types. All patients in our study underwent posterior approach open-window debridement and joint fusion by autologous iliac crest graft. Focal debridement using a posterior approach has the following advantages: First, it is easy to fully expose the sacroiliac joint due to its shallow position from the back side. A clear operative field permits operation under direct vision. Second, in front of the sacroiliac joint, there are ureter and iliac vessels across the front of the psoas and the femoral nerve between the psoas and iliacus. Using anterior debridement and bone graft carries potential risks of side injury. A posterior approach provides a more secure pathway [20].

Anterior abscesses should be disposed of as clean as possible in case of recurrence. Residual abscesses could be potential pathogens of recurrence. Because pus from abscesses yielded positive results for mycobacterium culture and acid-fast bacilli in other literature. [21] In our study, we got the similar positive results in one of the patients with abscess. In addition, according to Kim’s suggestion [10], abscess formation is one of the indications of surgical treatment of SJT. Curettage of pus and necrotic tissue may increase the effectiveness of chemotherapy and reduce the duration of treatment. And Papagelopoulos et al. [5] held the opinion that abscesses in the cases of progression or persistence during anti-tuberculostatic treatment, surgery should be involved. Because tuberculous abscess may form fistulization and spread to other place (even to the hip joint).

In order to manage anterior abscesses, supplementary one-stage anterior approach abscess clearance by curettage combined drainage may acquire satisfactory results. Additionally, anterior abscesses debridement should be done before posterior debridement and drain tubes should be placed to ensure valid drainage of the possible pus coming from the posterior joint when changing the patient’s posture from supine to prone position during operation.

Type C patients, that is, cases combined with spinal tuberculosis, can be supplied with one-stage debridement spontaneously. There would be no harmful mutual effects. Patients could achieve pain relief and solid bone fusion in both the sacroiliac joint and intervertebral body(Fig. 3f).

In previous studies, bone graft after debridement was absent in some patients [9, 10, 18, 22]. However, in our opinion, if the sacroiliac joints are severely destructed, it will generate an extensive joint defect after focal debridement which may cause instability and nonunion healing of the sacroiliac joints. Although tuberculosis can be eliminated, without solid bone healing and osseointegration, patients may suffer residual pain because of instability. In that case, patients cannot bear heavy loads or relatively intense daily activity. On account of that, all cases in our study underwent bone graft to ensure solid arthrodesis. Compared to the previous reports of surgical cases listed above, we achieved earlier joint fusion, no residual pain and almost complete functional improvement within 12 months. Additionally, there were no significant differences in VAS and ODI scores between 12 months and 24 months.

Furthermore, adequate, combined, disciplined and prolonged postoperative anti-tuberculosis chemotherapy is indispensable and important. It cannot be independently replaced by surgery. The clinical recovery criteria we applied was similar to Kim’s: no tenderness over the sacroiliac joint, no pain when bearing loads or working on daily activities, ESR returned to normal levels, clearance of lesions without recurrence and evident joint fusion. When patients met the criteria above, chemotherapy could be terminated, but a dosing time of no less than 12 months is recommended.

## Conclusions

SJT should be given adequate attention, even though it rarely occurs. The clinical manifestations and features of SJT are vague and non-specific. However, SJT could be accurately diagnosed by high vigilance and careful identification. CT and MRI are more sensitive in the early stage and can supplement routine laboratory examinations and X-ray. If SJT progresses to an advanced stage, posterior focal debridement and joint fusion with anterior clearance, if necessary, can achieve definite focus clearance and solid joint fusion. It can acquire full pain relief and satisfactory functional recovery and it a safe and efficient surgical method with a good safety profile.

## Additional file

Additional file 1: Figure S4. General pictures in the operation. (A) Body surface marker. (B-C) Debridement in operation. (JPEG 2847 kb)

## Abbreviations

Blood RT: Routine blood examination
EPTB: Extrapulmonary tuberculosis
ESR: Erythrocyte sedimentation rate
ODI: Oswestry Disability Index
PPD: Tuberculin purified protein derivative
SJT: Sacroiliac joint tuberculosis
TB: Tuberculosis
VAS: Visual analogue scale
