# Supplementary figures and images for: Sacroiliac joint tuberculosis: surgical management by posterior open-window focal debridement and joint fusion
Source: BMC Musculoskelet Disord. 2017 Nov 29;18:504. doi: 10.1186/s12891-017-1866-9 (PMC5708174; doi:10.1186/s12891-017-1866-9)

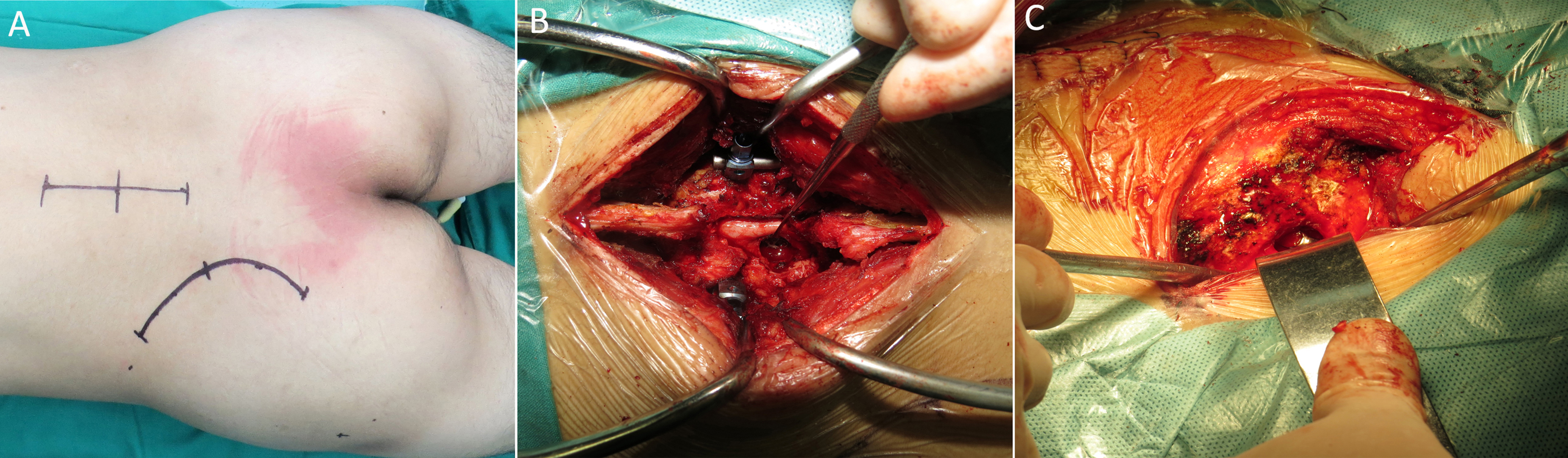

Supplement: Additional file 1: Figure S4. — General pictures in the operation. (A) Body surface marker. (B-C) Debridement in operation. (JPEG 2847 kb) [file 12891_2017_1866_MOESM1_ESM.jpg]
